# Efflux Pump Gene Expression in Multidrug-Resistant *Mycobacterium tuberculosis* Clinical Isolates

**DOI:** 10.1371/journal.pone.0119013

**Published:** 2015-02-19

**Authors:** Guilian Li, Jingrui Zhang, Qian Guo, Yi Jiang, Jianhao Wei, Li-li Zhao, Xiuqin Zhao, Jianxin Lu, Kanglin Wan

**Affiliations:** 1 Tuberculosis Branch, State Key Laboratory for Infectious Disease Prevention and Control, National Institute for Communicable Disease Control and Prevention, Chinese Center for Disease Control and Prevention, Beijing 102206, China; 2 Collaborative Innovation Center for Diagnosis and Treatment of Infectious Diseases, Hangzhou 310003, China; 3 Key Lab of Laboratory Medicine, Wenzhou Medical College, Wenzhou 325035, Zhejiang, China; 4 Laboratory, Shijiazhuang Gynecological and Obstetric Hospital, Shijiazhuang, Hebei, 050000, China; 5 Pathogenic Biology Institute, South of China University, Hengyang, Hunan 421001, China; Indian Institute of Science, INDIA

## Abstract

Isoniazid (INH) and rifampicin (RIF) are the two most effective drugs in tuberculosis therapy. Understanding the molecular mechanisms of resistance to these two drugs is essential to quickly diagnose multidrug-resistant (MDR) tuberculosis and extensive drug-resistant tuberculosis. Nine clinical *Mycobacterium tuberculosis* isolates resistant to only INH and RIF and 10 clinical pan-sensitive isolates were included to evaluate the expression of 20 putative drug efflux pump genes and sequence mutations in *rpoB* (RIF), *katG* (INH), the *inhA* promoter (INH), and *oxyR-ahpC* (INH). Nine and three MDR isolates were induced to overexpress efflux pump genes by INH and RIF, respectively. Eight and two efflux pump genes were induced to overexpress by INH and RIF in MDR isolates, respectively. *drrA*, *drrB*, *efpA*, *jefA (Rv2459)*, *mmr*, *Rv0849*, *Rv1634*, and *Rv1250* were overexpressed under INH or RIF stress. Most efflux pump genes were overexpressed under INH stress in a MDR isolates that carried the wild-type *katG*, *inhA*, and *oxyR-ahpC* associated with INH resistance than in those that carried mutations. The expression levels of 11 genes (*efpA*, *Rv0849*, *Rv1250*, *P55 (Rv1410c)*, *Rv1634*, *Rv2994*, *stp*, *Rv2459*, *pstB*, *drrA*, and *drrB*) without drug inducement were significantly higher (*P* < 0.05) in nine MDR isolates than in 10 pan-sensitive isolates. In conclusion, efflux pumps may play an important role in INH acquired resistance in MDR *M*. *tuberculosis*, especially in those strains having no mutations in genes associated with INH resistance; basal expression levels of some efflux pump genes are higher in MDR isolates than in pan-sensitive isolates and the basal expressional differences may be helpful to diagnose and treat resistant tuberculosis.

## Introduction

A number of mechanisms have been shown to be operative by which mycobacteria, especially *Mycobacterium tuberculosis*, develop resistance to anti-tuberculosis drugs. Mutations in target genes associated with the mode of action of these drugs have been considered the main mechanism for drug resistance in *M*. *tuberculosis* [1,2,3]. For example, >90% of rifampicin (RIF)-resistant *M*. *tuberculosis* isolates have at least one mutation in *rpoB*, which encodes the RNA polymerase subunit [1,4]. Of isoniazid (INH)-resistant isolates, 50–94% have at least one mutation in *katG*, which encodes catalase-peroxidase, 10–35% carry at least one mutation in the *inhA* promoter, and 10–40% carry at least one mutation in *oxyR-ahpC* [2,4,5,6,7,8]. Alternative mechanisms such as decreased cell wall permeability to drugs and active efflux pumping are likely to be important for conferring resistance in the isolates in which no target gene mutations are found [9,10,11,12,13].

*M*. *tuberculosis* has been studied for the presence and activity of a number of efflux pump genes and their encoded products. Most of the studies to date used laboratory strains and transferred hypothetical efflux genes into a heterologous host (*Mycobacterium smegmatis* or *Mycobacterium bovis*) and demonstrated that the overexpression of these genes increases resistance levels [11,12,13,14], whereas few studies investigated the relevance of active efflux in the drug resistance of clinical strains [15,16]. Some efflux pumps in drug-resistant *M*. *tuberculosis* are expressed at higher levels under drug stress [16,17]; however, low expression of efflux pump(s) from *Mycobacterium* in the absence of drug-induced stress has also been reported [18].

This study aimed to evaluate the expression of 20 putative efflux pump genes in multidrug-resistant (MDR) *M*. *tuberculosis* clinical isolates. These genes were annotated as probable/hypothetical drug efflux genes in the *M*. *tuberculosis* genome [19] and are available at the website of the Wellcome Trust Sanger Institute (www.sanger.ac.uk/Projects/M_tuberculosis/Gene_list/functional_classes/III.A.6.shtml). The expression of these 20 genes in MDR *M*. *tuberculosis* clinical isolates was examined previously, as was their contribution to RIF resistance in *M*. *tuberculosis* clinical isolates [20]. The study will further increase our understanding of the mechanism of active efflux in the INH and RIF resistance of *M*. *tuberculosis* and is the first investigation of the expressional differences between MDR clinical isolates and pan-sensitive clinical isolates.

## Materials and Methods

### Ethics statement

This study was approved by the ethics committee of the National Institute for Communicable Disease Control and Prevention, Chinese Center for Disease Control and Prevention. All patients involved in the study provided written informed consent.

### Antibiotics and chemicals

Middlebrook 7H9 broth and albumin-dextrose-catalase (ADC) supplement were purchased from Difco (Detroit, MI, USA). INH, RIF, ethambutol (EMB), streptomycin (STR), ofloxacin (OFX), kanamycin (KAN), capreomycin (CPM), amikacin (AK), carbonyl cyanide m-chlorophenylhydrazone (CCCP), and reserpine were purchased from Sigma-Aldrich (St. Louis, MO, USA). All solutions were prepared on the day of the experiment. Trizol was purchased from Invitrogen (Carlsbad, CA, USA). Alamar blue was obtained from AbD Serotec (Oxford, UK).

### Bacterial strains and drug susceptibility testing

Nine MDR isolates with different mutations in *katG*, *inhA*, *oxyR-ahpC*, and *rpoB*; 10 pan-sensitive isolates; and H37Rv were included. Seven MDR isolates were from Tibet Province, and two MDR isolates and 10 pan-sensitive isolates were from Fujian Province, China. All of the clinical isolates were recovered from a sputum specimen of patients with pulmonary tuberculosis and confirmed as the Beijing family by Spoligotyping as previously described by Kamerbeek et al. [21]. The isolate profiles of drug susceptibility were evaluated by the proportional method using Lowenstein-Jensen slants with the following: INH, 0.2 μg/mL; RIF, 40 μg/mL; STR, 4 μg/mL; EMB, 2 μg/mL; KAN, 30 μg/mL; OFX, 2 μg/mL; CPM, 40 μg/mL [22]; and AK, 30 μg/mL [23]. Nine MDR isolates were resistant to only INH and RIF but were sensitive to the other six drugs, while the 10 pan-sensitive isolates were sensitive to all eight drugs above.

### Determination of minimum inhibitory concentrations (MICs)

A microplate alamar blue assay was performed as previously described [24] to determine the MICs of INH and RIF of the 19 clinical isolates. H37Rv was used as a sensitive control. The effect of the efflux inhibitors CCCP and reserpine on the MICs of these antibiotics was also studied. Two-fold serial dilutions of INH or RIF were made directly in the wells including or excluding 0.5 μg/mL CCCP [12,17] or 5 μg/mL reserpine [17]. Drug concentrations included 0.001–128 μg/mL INH or 0.001–256 μg/mL RIF. All tests were conducted twice. The MIC was defined as the lowest drug concentration that prevented a color change. Isolates with MICs of INH <0.25 μg/mL and RIF ≤1 μg/mL were defined as being sensitive to INH and RIF, respectively [24].

### RNA extraction and reverse transcription

All 19 *M*. *tuberculosis* strains were subcultured in 7H9 medium with ADC supplement, and collected for RNA extraction. For the nine MDR isolates, RIF and INH were added to these cultures individually at subinhibitory concentrations (half of the MIC), incubated at 37°C for 25 days, and collected for RNA extraction. Total bacterial RNA was isolated using Trizol reagent according to the manufacturer’s instructions. The quality and integrity of the total RNA was assessed using a nanophotometer (Implen, Munden, Germany) and agarose gel electrophoresis. After treatment with DNase I (amp grade; Invitrogen), the lack of DNA contamination of the RNA samples was confirmed by polymerase chain reaction (PCR) amplification of *rpoB* directly from RNA. The forward and reverse primers are listed in S1 Table. A 50-μL aliquot of the PCR mixture (Kangwei Biotechnology, Beijing, China) was used, and the PCRs were denatured at 94°C for 5 min and subjected to 35 cycles at 94°C for 30 s, 62°C for 30 s, and 72°C for 30 s, followed by a final extension at 72°C for 10 min. RNA (1.5 μg) was reverse transcribed according to the manufacturer’s recommendations (Transgen Bio-Technology Company, Beijing, China), and the thermal cycling conditions were as follows: 25°C for 10 min, 42°C for 60 min, and 85°C for 5 min. The cDNA was maintained at −20°C. Two cDNA preparations were made for each strain.

### Quantification of gene expression using real-time quantitative PCR (qPCR)

The primers of the 20 genes are described in S2 Table. The assay was performed using a qPCR kit (Transgen Bio-Technology Company) in a CFX96 thermocycler (Bio-Rad Laboratories Inc., Hercules, CA, USA). Briefly, each 0.2-mL tube contained 10 μL 2× qPCR mix, 3 pmol each primer, 10 ng cDNA, and RNase-free water to a final volume of 20 μL. The thermal cycling conditions were as follows: 50°C for 2 min and 95°C for 8 min, then 45 cycles of denaturation at 94°C for 10 s, annealing at 59°C for 15 s, and extension at 72°C for 20 s, and the last step consisted of a melting curve analysis (65–95°C).

The fold change in the expression of genes under drug stress in nine MDR isolates was calculated by 2^-ΔΔCT^ method of Livak and Schmittgen [25]. When compared with the non-exposed control genes showing expression levels above 1 were considered to be increased; equal to or above four were considered to be overexpressed [25,26,27,28]. The expression levels of 19 isolates without drug stress was caculated by 2^-ΔCT^ method. The difference in threshold cycle (ΔCT) values were obtained by subtracting the CT value of *polA* from the CT value obtained for each gene. The ΔΔCT values were obtained by subtracting the ΔCT value obtained for each gene without drug inducement from the ΔCT value of genes induced by INH/RIF stress. qPCR was performed three times for each gene of each cDNA, and a mean expression level of each gene of one isolate was obtained from two cDNA preparations. *polA* is a housekeeping gene that is expressed at a stable level in the isolates and can used as an internal invariant control. The expression levels of the 20 genes without drug inducement of the nine MDR isolates were compared with those of the 10 pan-sensitive isolates.

### DNA extraction and sequencing analysis

Twenty strains cultured on solid Lowenstein-Jensen medium were harvested and killed by heating at 80°C for 90 min. The genomic DNA was extracted by the cetyltrimethylammonium bromide method.

The sequences of genes including *rpoB* for RIF, *katG* and *inhA* regulator sequence, and *oxyR-ahpC* genes for INH (reported to carry major mutations associated with RIF and INH resistance) were analyzed by PCR and DNA sequencing. The sequences of the specific primers and the sizes of the amplicons are presented in S1 Table. The PCR conditions of four genes were identical and described in RNA extraction and reverse transcription section. Partial PCR products were characterized by DNA sequencing using the forward primers on an ABI Prism 3730 automated DNA sequencer (ABI Prism). The resulting DNA sequences were analyzed using the basic local alignment search tool (http://www.ncbi.nih.gov/BLAST), and the specific mutations in protein sequences of the individual isolates were identified.

### Data analysis

SPSS 16.0 (SPSS Inc., Chicago, IL, USA) was used to perform Mann-Whitney test, and the difference was considered to be statistically significant when *P* < 0.05.

## Results and Discussion

### INH/RIF MICs and the effect of efflux pump inhibitors on INH/RIF MICs of pan-sensitive clinical isolates

As shown in Table 1, the INH MICs for the 10 pan-sensitive clinical isolates and H37Rv were 0.016–0.063 μg/mL, whereas the RIF MICs were 0.0078–0.25 μg/mL. No decreases in the INH and RIF MICs were observed in the presence of the efflux pump inhibitors CCCP (0.5 μg/mL) or reserpine (5 μg/mL) in these isolates, which supported the results of an earlier study [18].

**Table 1:** Minimum inhibitory concentrations (MICs) for isoniazid (INH) and rifampicin (RIF) in the absence or presence of efflux inhibitors and the number of genes overexpressed in nine multidrug-resistant (MDR) *M*. *tuberculosis* isolates.

Isolate no.	MIC (μg/mL) of INH +			Genes overexpressed under INH stress	MIC (μg/mL) of RIF +			Genes overexpressed under RIF stress
	None	CCCP	RSP		None	CCCP	RSP	
CCDC09103	4	4	**2**	*drrA*, *efpA*, *jefA* (*Rv245*9)	64	64	64	no
CCDC05148	4	4	4	*drrA*	256	256	256	no
CCDC09078	2	2	2	*drrA*, *mmr*	>256	**32**	**32**	*drrB*
CCDC10223	1	**0.25**	**0.25**	*drrA*, *drrB*, *efpA*, *Rv0849*, *jefA* (*Rv245*9)	128	**64**	**64**	no
CCDC10038	1	**0.25**	**0.5**	*drrA*, *drrB*, *efpA*, *jefA* (*Rv245*9)	256	**64**	**64**	*Rv1634*
CCDC10138	1	**0.25**	**0.125**	*drrA*, *drrB*, *efpA*, *jefA* (*Rv245*9)	64	64	64	no
CCDC10017	>128	128	>128	*drrA*	32	**16**	32	no
CCDC10143	1	1	**0.5**	*drrA*, *jefA* (*Rv245*9)	1	**0.25**	**0.25**	no
CCDC10219	>128	**64**	**16**	*drrA*, *drrB*, *efpA*, *jefA* (*Rv245*9), *Rv1250*, *Rv1634*	32	**4**	32	*drrB*
CCDC10328	0.031	0.031	0.031	ND	0.0625	0.0625	0.0625	ND
CCDC10330	0.031	0.031	0.031	ND	0.125	0.125	0.125	ND
CCDC10201	0.063	0.063	0.063	ND	0.25	0.25	0.25	ND
CCDC10231	0.063	0.063	0.063	ND	0.0625	0.0625	0.0625	ND
CCDC10246	0.063	0.063	0.063	ND	0.125	0.125	0.125	ND
CCDC10255	0.063	0.063	0.063	ND	0.0078	0.0078	0.0078	ND
CCDC10261	0.063	0.063	0.063	ND	0.125	0.125	0.125	ND
CCDC10293	0.016	0.016	0.016	ND	0.125	0.125	0.125	ND
CCDC10238	0.063	0.063	0.063	ND	0.0625	0.0625	0.0625	ND
CCDC10239	0.031	0.031	0.031	ND	0.0625	0.0625	0.0625	ND
H37Rv	0.063	0.063	0.063	ND	0.0625	0.0625	0.0625	ND

CCCP, carbonyl cyanide m-chlorophenylhydrazone; RSP, reserpine; numbers in bold mean that the MIC decreased relative to the INH or RIF MIC without inhibitors. ND, not determined.

### INH/RIF MICs and the effect of efflux pump inhibitors on INH/RIF MICs of MDR clinical isolates

As demonstrated in Table 1, the INH MICs of nine MDR isolates varied from 1 to >128 μg/mL, whereas the RIF MICs varied from 1 to >256 μg/mL. Of the nine MDR isolates cultured in the presence of efflux pump inhibitors CCCP (0.5 μg/mL) or reserpine (5 μg/mL), four isolates (44.4%) and six isolates (66.7%) demonstrated a significant decrease in the INH MIC after CCCP and reserpine exposure, respectively. Additionally, six isolates (66.7%) and four isolates (44.4%) showed a decrease in the RIF MICs after exposure to CCCP and reserpine, respectively. Sensitivity to INH was fully restored (MIC <0.25 μg/mL) in one isolate (CCDC10138) after reserpine exposure, whereas the RIF sensitivity was fully restored (MIC ≤1 μg/mL) in an additional isolate (CCDC10143) after exposure to reserpine and CCCP. In total, the MICs of INH or RIF of nine MDR isolates except one were affected by CCCP and reserpine. Mann-Whitney test showed that the fold change of MIC of RIF or INH of MDR strains decreased by CCCP or reserpine were all significantly higher than pan-sensitive group (*P* values were all <0.05), suggesting that the INH and RIF resistance levels could be affected by efflux pump inhibitor exposure in resistant strains, which is consistent with the reports of Rodrigues et al. [27] and Escribano et al. [29]. Rodrigues et al [27] found that the INH MIC of one INH-induced *M*. *bovis BCG* strain was decreased four- and two-fold by the drug efflux inhibitors chlorpromazine and thioridazine, respectively, and the INH MICs of three INH-induced *M*. *tuberculosis* isolates decreased more than 32-fold. Escribano et al. [29] observed that the MIC of fluoroquinolones in four to seven of seven fluoroquinolone-resistant *M*. *tuberculosis* clinical isolates was decreased by inhibitors MC 207.110 (Phe-Arg-β-naphthylamide) and reserpine. A previous study reported that BALB/c mice treated with first-line drugs in combination with the efflux pump inhibitor verapamil could significantly reduce pulmonary colony-forming units after 1 and 2 months of treatment (*P* < 0.05) [30]. These results suggest that efflux pump inhibitors have a potential use in tuberculosis treatment.

### Expression of efflux pump genes under INH or RIF stress in MDR isolates

As demonstrated in Tables 1 and 2, all nine MDR isolates had at least one gene that was overexpressed under INH or RIF stress. More strains were induced to overexpress efflux pump genes by INH than by RIF (nine vs. three), and more genes were induced by INH than by RIF (eight vs. two). Additionally, more drug efflux pumps were overexpressed in MDR isolates in which the INH or RIF MICs decreased more than four-fold after CCCP or reserpine exposure under INH or RIF stress.

Our analysis demonstrated that INH/RIF induced differential expression of some of these genes, see Table 2. *drrA*, *drrB*, *mmr*, *efpA*, *jefA* (*Rv2459*), *Rv0849*, *Rv1634*, and *Rv1250* were overexpressed (more than four-fold change). *drrABC* is a putative doxorubicin-resistance operon in *M*. *tuberculosis* [19]. *drrAB* expressed in *M*. *smegmatis* conferred resistance to a broad range of clinically relevant antibiotics, including EMB, STR, norfloxacin, erythromycin, tetracycline, and chloramphenicol [11]. In this study, *drrA* was overexpressed in all nine MDR isolates induced by INH and zero MDR isolates induced by RIF, suggesting that *drrA* was one of the factors for INH resistance in *M*. *tuberculosis*. Pang et al. reported that *drrA* may be responsible for low-level resistance to RIF [20], which was not consistent with our results. *drrB* was up-regulated consistently upon INH/RIF treatment in only those MDR strains that showed a significant reduction in MIC with CCCP/reserpine in this study, suggesting that *drrB* plays an important role in INH/RIF resistance in *M*. *tuberculosis*.

**Table 2:** Differential expression of efflux pump genes under isoniazid or rifampicin stress in *M*. *tuberculosis* by real-time reverse transcription quantitative PCR.

Stress condition	Gene	*No*. of MDR isolates with different fold change (2^-ΔΔCT^ value, n = 9)*				
		0-	1-	2-	3-	>4-
Isoniazid	*drrA*	0	0	0	0	9
	*drrB*	0	2	1	2	4
	*efpA*	0	0	2	2	5
	*mmr*	0	2	2	4	1
	*emrB*	2	6	1	0	0
	*Rv0849*	0	2	4	2	1
	*mmpL13a*	3	5	1	0	0
	*mmpL13b*	4	4	1	0	0
	*Rv1250*	0	3	2	3	1
	*tap* (*Rv1258c*)	3	4	2	0	0
	*P55* (*Rv1410c*)	1	4	2	2	0
	*Rv1634*	0	2	5	1	1
	*Rv2994*	1	5	3	0	0
	*stp*	2	6	1	0	0
	*jefA* (*Rv245*9)	0	0	1	2	6
	*pstB*	4	3	2	0	0
	*Rv2456*	2	7	0	0	0
	*Rv2265*	3	6	0	0	0
	*Rv3239*	4	5	0	0	0
	*Rv1877*	2	7	0	0	0
Rifampicin	*drrA*	4	5	0	0	0
	*drrB*	0	0	3	4	2
	*efpA*	0	6	2	1	0
	*mmr*	2	7	0	0	0
	*emrB*	3	5	1	0	0
	*Rv0849*	1	6	2	0	0
	*mmpL13a*	6	2	1	0	0
	*mmpL13b*	4	5	0	0	0
	*Rv1250*	3	5	1	0	0
	*tap* (*Rv1258c*)	2	6	1	0	0
	*P55* (*Rv1410c*)	1	5	3	0	0
	*Rv1634*	0	1	4	3	1
	*Rv2994*	2	4	3	0	0
	*stp*	0	0	6	3	0
	*jefA* (*Rv245*9)	2	5	2	0	0
	*pstB*	2	4	3	0	0
	*Rv2456*	4	5	0	0	0
	*Rv2265*	5	4	0	0	0
	*Rv3239c*	5	4	0	0	0
	*Rv1877*	3	5	0	0	0

* genes showing expression levels >one-fold change were considered to be increased and ≥ four-fold change were considered to be overexpressed under INH/RIF stress.

Previous reports indicated that the expression of *efpA* increased in response to isoniazid [16,31], thiolactomycin [32], isoxyl, tetrahydrolipstatin, and three compounds from the Southern Research Institute, Birmingham, AL, USA [33]. Fu [31] and Gupta et al. [16] showed that the induced *efpA* expression was 4- and 4.5-fold greater under INH stress, respectively, which was consistent with the results in our study. *jefA* (*Rv245*9) has been reported to respond to INH stress, and Gupta et al. found that the increased transcription of *jefA* (*Rv245*9) leads to increased resistance to EMB and INH in *M*. *tuberculosis* [16,17]. Mmr has been reported to mediate the efflux of different chemical classes and antibiotics [34]. Expression of the *M*. *tuberculosis* protein Mmr in *M*. *smegmatis* was shown to resistance to tetraphenylphosphonium, erythromycin, ethidium bromide, acriflavine, safranin O, and pyronin Y [35]. Gupta et al. [16] found that *mmr* had two-fold higher expression in two isolates under the INH-stressed condition and three-fold higher expression in three isolates under the levofloxacin-stressed condition. Rv1634 is a member of the major facilitator superfamily in *M*.*tuberculosis*, similar to many antibiotic resistance (efflux) proteins, including DTDP-glucose dehydratase from *Streptomyces violaceoruber* [19,36,37,38]. Rv1634 has been reported to decrease susceptibility to various fluoroquinolones when overexpressed in *M*. *smegmatis* and involve in norfloxacin and ciprofloxacin efflux [37]. Louw et al. reported that Rv1634 was upregulated in strains with a Beijing genotype after exposure to RIF for 24 h [30]. Balganesh et al. [34] reported that the efflux pump encoded by *Rv0849* mediates the efflux of RIF and AK. Rv1250 is a probable drug-transport integral membrane protein having 579 amino acid (aa) and highly similar to the tetracenomycin C protein from *Streptomyces glaucescen* (32.9% identity in 517 aa overlap). Rv1250 is also similar to Rv3239C from *M*. *tuberculosis* (31.9% identity in 423 aa overlap) [19]. No further information on this protein has been reported.

Together, these results indicate that more efflux pumps may respond to INH than RIF in *M*. *tuberculosis*. A possible explanation for this may be that INH exerts its bactericidal activity by attacking the cell wall mycolic acid after being converted to a range of oxygenated and organic toxic radicals by bifunctional bacterial enzyme catalase-peroxidase (KatG) [39,40], whereas RIF acts by arresting DNA-directed RNA synthesis in the cytoplasm of *M*. *tuberculosis* [41,42]. Efflux pumps are located in the membrane of *M*.*tuberculosis* [19,37,43]. While the cell wall of *M*.*tuberculosis* is attacked by non-lethal dose of INH, more efflux pumps or combinations thereof are induced to efflux INH to avoid *M*.*tuberculosis* being killed.

In 10 pan-sensitive isolates, none of 20 genes were induced to overexpress by RIF and INH in our previous study [44], which was consistent with the results that no decreases in the INH and RIF MICs in the presence of the efflux pump inhibitors CCCP (0.5 μg/mL) or reserpine (5 μg/mL) in these isolates.

### Efflux pump gene expression in MDR isolates carrying gene mutations

Mutations in drug target genes are still thought to be the principal mechanism of drug resistance. Three (CCDC09103, CCDC05148, and CCDC09078) of the nine MDR isolates carried mutations of *katG* 315 + *rpoB* 531. Two isolates (CCDC10038 and CCDC10223) carried mutations of *katG* 298 + *inhA* (-15) + *rpoB* 531. One isolate (CCDC10138) carried mutations of *katG* 222 + *rpoB* 531. One isolate (CCDC10017) carried mutations of *ahpC* (-10) + *rpoB* 490 + *rpoB* 531 + *rpoB* 559. One isolate (CCDC10143) carried mutations of *inhA* (-15) + *rpoB* 511 + *rpoB* 526. One isolate (CCDC10219) carried a deletion mutation that affected codons 516 and 517 of *rpoB* and had the wild-type *katG*, *inhA* promoter, and *oxyR-ahpC* (Table 3). Eight of nine MDR isolates overexpressed at least one efflux pump gene, suggesting that efflux pump genes may be involved in resistance to INH and RIF in addition to the mutations in related genes [45]. Isolate CCDC10219 carried a deletion mutation in *rpoB* and had the wild-type version of genes associated with INH resistance; most efflux pump genes (six genes) were overexpressed compared with those in other isolates under INH stress, suggesting that efflux pump gene expression was the major cause of INH resistance in this isolate.

**Table 3:** Characteristics of mutations of 9 MDR ***M*. *tuberculosis*** isolates.

Isolate no.	Gene mutations			
	*rpoB*	*katG*	*inhA*	*oxyR-ahpC*
CCDC09103	531TCG-TTG	315AGC-ACC	wt	wt
CCDC05148	531TCG-TTG	315AGC-ACC	wt	wt
CCDC09078	531TCG-TTG	315AGC-ACC	wt	wt
CCDC10223	531TCG-TTG	298TTG-TGG	(-15)C-T	wt
CCDC10038	531TCG-TTG	298TTG-TGG	(-15)C-T	wt
CCDC10138	531TCG-TTG	222GCG-GAG	wt	wt
CCDC10017	531TCG-TTG,490CAG-CGG, 559ATC-ACC	wt	wt	(-10)C-T
CCDC10143	511CTG-CCG,526CAC-CAG	wt	(-15)C-T	wt
CCDC10219	CCA deleted in 76838–76840 (BX842574.1)	wt	wt	wt

wt, wild type.

### The differences in drug efflux pump gene expression between MDR isolates and pan-sensitive isolates

The quantification results of the expression levels of 20 genes in nine MDR isolates and 10 pan-sensitive isolates are shown in S3 Table. In seven MDR strains CCDC09103, CCDC09078, CCDC10138, CCDC10143, CCDC10223, CCDC10017, and CCDC05148, gene *Rv2265* showed the lowest expression levels from 0.014 to 0.246, and *jefA* (*Rv2459*) showed the highest expression levels from 8.744 to 33.063 except that *efpA* showed the highest expression of 6.329 in strain CCDC05148. In MDR strain CCDC10038 and CCDC10219, *Rv3239c* showed the lowest expression levels which were 0.242 and 0.174, respectively, and *drrA* showed the highest expression levels which were 6.493 and 65.983, respectively. The mean expression levels of *efpA*, *Rv1250*, *jefA* (*Rv245*9), and *drrA* in MDR isolates were all higher than 5.0 without any drug inducement.

Of ten pan-sensitive clinical isolates, *drrA* showed the highest expression levels from 1.493 to 5.919 in eight isolates, while *efpA* shown highest expression level of 10.801 in strain CCDC10246 and *Rv1634* showed the highest expression level of 3.205 in strain CCDC10293. In four pan-sensitive clinical isolates CCDC10330, CCDC10201, CCDC10261, and CCDC10293, *mmpl13b* showed the lowest expression levels from 0.113 to 0.159. In six pan-sensitive clinical isolates CCDC10328, CCDC10238, CCDC10239, CCDC10231, CCDC10246, and CCDC10255, *Rv3239c* showed the lowested expression levels from 0.060 to 0.178.

In this study, we examined the expressional differences between MDR isolates and pan-sensitive isolates without drug inducement. The expression levels of 11 genes (*efpA*, *Rv0849*, *Rv1250*, *P55* (*Rv1410c*), *Rv1634*, *Rv2994*, *stp*, *Rv2459*, *pstB*, *drrA*, and *drrB*) were significantly higher (*P* < 0.05) in nine MDR isolates than in 10 pan-sensitive isolates (Table 4). We also found that the expression of three genes (*Rv1877*, *Rv2265*, and *mmpL13a*) was significantly lower (*P* < 0.05) in nine MDR isolates than in 10 pan-sensitive isolates. Li et al. reported that deleting the closest homologs of *mmpL13a-mmpL13b* (Rv1145-Rv1146) in *M*. *smegmatis* did not alter the drug susceptibility, whereas deleting the *Rv1877* homolog resulted in increased susceptibility to ethidium bromide, acriflavine, and erythromycin [38]. These results suggested that the role of these efflux pumps is probably not to pump out anti-tubercular drugs but, rather, that they regulate the intracellular levels of nutrients and co-factors. The differences between MDR and pan-sensitive isolates suggest that quantifying the expression levels of *M*. *tuberculosis* efflux pump genes may be a new method to diagnose resistant tuberculosis and decide whether combining efflux pump inhibitors to anti-tubercular drugs would be effective to treat resistant tuberculosis.

**Table 4:** Relative expression levels (2^-ΔCT^ value) of drug efflux genes of nine MDR *M*. *tuberculosis* isolates and 10 pan-sensitive isolates.

Gene	Pan-sensitive group		MDR group	
	Median value	25%–75% value	Median value	25%–75% value
*efpA* ^a^	1.39	0.56–2.54	6.49	5.29–11.06
*emrB*	0.96	0.81–1.09	0.79	0.64–1.45
*Rv0849* ^a^	0.37	0.27–0.28	1.58	0.94–2.73
*Rv1250* ^a^	1.91	0.80–3.43	7.74	5.04–10.00
*tap* (*Rv1258c*)	0.76	0.65–1.39	1.15	0.41–1.34
*P55* (*Rv1410c*)^a^	0.98	0.64–1.30	2.01	1.62–5.51
*Rv1634* ^b^	0.36	0.25–0.43	0.54	0.40–1.69
*Rv2994* ^a^	0.47	0.37–0.56	1.45	0.89–2.22
*Rv1877* ^b^	0.55	0.48–0.72	0.34	0.18–0.49
*stp* ^a^	0.68	0.44–0.78	1.30	1.21–3.70
*jefA* (*Rv245*9)^a^	0.93	0.78–1.12	5.37	4.42–19.32
*Rv2265* ^a^	0.34	0.24–0.52	0.06	0.04–0.27
*Rv2456*	0.48	0.32–0.68	0.21	0.14–0.53
*Rv3239*	0.14	0.11–0.17	0.17	0.06–0.22
*mmpL13a* ^b^	0.52	0.29–0.69	0.20	0.10–0.50
*mmpL13b*	0.17	0.13–0.23	0.20	0.16–0.41
*pstB* ^a^	0.65	0.55–1.15	3.29	2.26–5.98
*drrA* ^a^	3.52	1.54–5.24	9.46	6.06–15.22
*drrB* ^a^	1.12	0.79–1.50	3.81	2.83–7.12
*mmr*	0.23	0.19–0.29	0.35	0.23–0.47

^a^Significant differences between the MDR and pan-sensitive groups (*P* < 0.01)

^b^Significant differences between the MDR and pan-sensitive groups (*P* < 0.05).

### Conclusion

Efflux pumps may play an important role in INH acquired resistance in MDR *M*. *tuberculosis*, especially in strains without mutations in *katG*, *inhA*, and *oxyR-ahpC*, which are associated with INH resistance. The basal expressional differences of some drug efflux pump genes between MDR and pan-sensitive isolates may be helpful to diagnose and treat resistant tuberculosis. However, we acknowledge that we were unable to demonstrate a direct relationship between the resistance level of INH and/or RIF and the activation of specific genes. Further efforts are required to elucidate the actual roles of drug efflux pumps in the drug resistance of *M*. *tuberculosis*.

## Supporting Information

S1 Table Primers used to amplify and sequence *rpoB*, *katG*, *inhA*, and *oxyR-ahpC* mutations. (DOCX)

S2 Table Primers used in this study to quantify gene expression. (DOCX)

S3 Table The relative expression levels (2^-△CT^ value) of each gene of 20 strains. (XLSX)
