# Supplementary material for: Efflux Pump Gene Expression in Multidrug-Resistant Mycobacterium tuberculosis Clinical Isolates
Source: PLoS One. 2015 Feb 19;10(2):e0119013. doi: 10.1371/journal.pone.0119013 (PMC4335044; doi:10.1371/journal.pone.0119013)
Supplement: S1 Table — (DOCX) [file pone.0119013.s001.docx]

S1_Table. Primers used to amplify and sequence *rpoB, katG*, *inhA*, and *oxyR-ahpC* mutations

| Gene (accession no.) | Primer | Sequence (5′→3′) | Amplicon size (bp) |
| --- | --- | --- | --- |
| *rpoB* (BX842574.1) | F | ACCGACGACATCGACCACTT | 450 |
|  | R | GTACGGCGTTTCGATGAACC |  |
| *katG* (X68081) | F | AATCGATGGGCTTCAAGACG | 500 |
|  | R | CTCGTAGCCGTACAGGATCTCG |  |
| *inhA* (BX842576) | F | CCTCGCTGCCCAGAAAGGGA | 248 |
|  | R | ATCCCCCGGTTTCCTCCGGT |  |
| *oxyR-ahpC* (BX842579.1) | F | GAGACCGGCTTCCGACCACC | 293 |
|  | R | GCTGGTAGGCGGGGAATTGAT |  |

F, forward primer; R, reverse primer
