# Supplementary material for: Efflux Pump Gene Expression in Multidrug-Resistant Mycobacterium tuberculosis Clinical Isolates
Source: PLoS One. 2015 Feb 19;10(2):e0119013. doi: 10.1371/journal.pone.0119013 (PMC4335044; doi:10.1371/journal.pone.0119013)
Supplement: S2 Table — (DOCX) [file pone.0119013.s002.docx]

S2_Table. Primers used in this study to quantify gene expression

| No. | Gene | Primer sequence (5′→3′) | Amplicon size (bp) |
| --- | --- | --- | --- |
| 1 | *efpA* | CGCCCTACGGGAAACCAACAAAGA | 226 |
|  |  | GCGGAACAAGTGGAACGGCACGAC |  |
| 2 | *emrB* | ACCGCACAGAACATCCGCTCATAG | 148 |
|  |  | GATTGGTGCAACACTTGCTGGAGG |  |
| 3 | *Rv0849* | GTCGTTCGCAACCGTCCGTTTCTG | 94 |
|  |  | CCTGCATGGGCAGAGCCAGATAGA |  |
| 4 | *Rv1250* | GCAGCCTTGGATTTGGGCGGTGAT | 133 |
|  |  | GGACAAGCTGAAGTTCCGGTCGTT |  |
| 5 | *tap(Rv1258c)* | CGTCTGGAACCTGCGGGTATTGCG | 118 |
|  |  | CGGTTGCTGGTGGTCGGTGAAGTA |  |
| 6 | *P55(Rv1410c)* | ATCCCGACGGCAAACACGTACTGC | 205 |
|  |  | ACATCAACCAGCGTCACCATCAGC |  |
| 7 | *Rv1634* | TCGATACCTACGTGCCGCTGTTCG | 157 |
|  |  | GCTGCCACGACATGCCCGATAACT |  |
| 8 | *Rv2994* | ATGCGTCCCGTCCGCCTGAT | 147 |
|  |  | GGTGGCTTCTAGCCCGTTGTCC |  |
| 9 | *Rv1877* | TGTGCTGCGTCCTGTCCTTCGT | 235 |
|  |  | AGGAACGCAACCGCCATCAG |  |
| 10 | *stp* | TCCGATGATGGATCTGACCCTG | 217 |
|  |  | GCCAACCAGGTGCCCAACA |  |
| 11 | *jefA* (*Rv245*9) | CGTCGCCCTGATCGCATACA | 213 |
|  |  | CAGGACATCACCACGAAGTAGACG |  |
| 12 | *Rv2265* | CGGTTGTCCTCGGTAATCCT | 112 |
|  |  | AACCCGAACGTGCCAAAC |  |
| 13 | *Rv2456c* | CAGCGAACCCCACCAAA | 140 |
|  |  | GCACAATCGAGACGAAGGAA |  |
| 14 | *Rv3239c* | GCCGATTCCTGGCACTTTT | 146 |
|  |  | ATGTGGATGGCGGTGTGTT |  |
| 15 | *mmpL13a* | GACGACCTGCTGGTGATGGAGTTG | 242 |
|  |  | CGACTGACGATGAGCAGCGTGTAG |  |
| 16 | *mmpL13b* | ATGTTCGGCCTCGGCCTGACTTTA | 182 |
|  |  | GAACGTCTCCTCGAAACCGGCTCT |  |
| 17 | *pstB* | CTGGACCCGACTACCACCGAGAA | 95 |
|  |  | GCCTGGGCAAGGTTATGGGTC |  |
| 18 | *drrA* | TAGACATCGCGTGCGGATTGGT | 147 |
|  |  | GCGTGGTCAACAACGTGGCAAT |  |
| 19 | *drrB* | TCGCCAGCAACTTAGGGCAATACA | 233 |
|  |  | TCCGATGACGTAGCCGCAAACTAG |  |
| 20 | *mmr* | TAGTGGGTTATGGCATCGCTTTCG | 167 |
|  |  | GACGCCAACCACCTTCATCACAGA |  |
| 21 | *polA* | GTCGTGGTTGGACCTTGGAGGG | 181 |
|  |  | GCGTCCGTATCGTCGTCATCG |  |
